# Differential gene expression analysis reveals novel genes and pathways in pediatric septic shock patients

**DOI:** 10.1038/s41598-019-47703-6

**Published:** 2019-08-02

**Authors:** Akram Mohammed, Yan Cui, Valeria R. Mas, Rishikesan Kamaleswaran

**Affiliations:** University of Tennessee Health Science Center, Memphis, TN USA

**Keywords:** Predictive markers, Microarrays

## Abstract

Septic shock is a devastating health condition caused by uncontrolled sepsis. Advancements in high-throughput sequencing techniques have increased the number of potential genetic biomarkers under review. Multiple genetic markers and functional pathways play a part in development and progression of pediatric septic shock. We identified 53 differentially expressed pediatric septic shock biomarkers using gene expression data sampled from 181 patients admitted to the pediatric intensive care unit within the first 24 hours of their admission. The gene expression signatures showed discriminatory power between pediatric septic shock survivors and nonsurvivor types. Using functional enrichment analysis of differentially expressed genes, we validated the known genes and pathways in septic shock and identified the unexplored septic shock-related genes and functional groups. Differential gene expression analysis revealed the genes involved in the immune response, chemokine-mediated signaling, neutrophil chemotaxis, and chemokine activity and distinguished the septic shock survivor from non-survivor. The identification of the septic shock gene biomarkers may facilitate in septic shock diagnosis, treatment, and prognosis.

## Introduction

Septic shock is a life-threatening organ dysfunction caused by an imbalanced host response to infection^1^. Multi-omics sequencing technologies have increased the number of genetic biomarkers^2^. Single or combination biomarkers are increasingly being analyzed and tested in the context of genes, RNA, or proteins^3–6^. Many strategies for uncovering biomarkers exist, such as mass-spectrometry, protein arrays, and gene-expression profiling. Furthermore, it has been demonstrated that multiple genes and immune system-related pathways participate in development of pediatric septic shock^7^.

High-throughput technologies support the analysis of gene expressions and also enable the ability to determined activity of these genes in different conditions^8^. Statistical testing and machine learning methods have been frequently used to successfully utilize omics data for biomarker discovery^2,9–18^.

The purpose of this study is to identify differentially expressed pediatric septic shock biomarkers using gene expression data to predict long-term outcomes. To that end, gene expression data from 181 samples collected from critically ill patients admitted to the pediatric intensive care unit (PICU) within the first 24 hours of their admission, were analyzed using multiple statistical testing methods to identify gene biomarkers. The gene expression profiles discovered by this statistical approach may lead to new insights that support sucessful prognosis, especially among patients with poor long term outcomes^19^. Furthermore, these approaches can augment parallel efforts using clinical markers for earlier identification of sepsis and septic shock, providing meaningful input at the point of care^20–22^. Using functional gene-set enrichment analysis, we validated the known septic shock-related genes, pathways and functional groups, and identified the unexplored septic shock-related genes and functional groups. Discovery of potential gene biomarkers may provide effective septic shock diagnosis, treatment, and prognosis.

## Results

The pediatric septic shock dataset contains gene expression profiles of peripheral blood samples collected from 181 patients including 154 survivors and 27 non-survivors within the first 24 hours of admission. Overall mean age of the patient cohort was 3.69 years with a standard deviation of 3.28. Mean age of the survivors was 3.78 years (standard deviation of 3.15), compared to 3.19 (standard deviation of 3.93) for the non-survivors. Males (109; 60.2%) had higher representation than females (72; 39.8%). Of the 27 non-survivors, 19 were male and only 8 were female; whereas, in the non-survivor group the male and females were 90 and 64, respectively. Clinical characteristics (such as mortality, organism, source of infection, malignancies, immunosuppression, gram-positive bacteria, and acute kidney injury) of the 181 patients are included in Supplementary Dataset 1.

Etiologic diagnosis was identified for 107 (59.12%) patients. As shown in Table 1, the most frequently identified pathogens were *Streptococcus* *pneumoniae* (in 17 cases), *Staphylococcus aureus* (in 17 cases), and *Streptococcus pyogenes* (in 12 cases). Detailed clinical characteristics including source of infection and organism are given in Supplementary Dataset 1.

**Table 1:** Etiology of septic shock in 181 patients.

Etiology	Number of patients	Total (%)
*Streptococcus pneumoniae*	17	9.39
*Staphylococcus aureus*	17	9.39
*Streptococcus* *pyogenes*	12	6.63
*Klebsiella*	9	4.97
*Streptococcus* *agalactiae*	7	3.87
*Neisseria meningitidis*	7	3.87
*Enterococcus*	5	2.76
*Gram-negative rods*	4	2.21
*Simplexvirus*	3	1.66
*influenza* *A* *virus*	3	1.66
Mixed	3	1.66
*Enterobacter*	2	1.11
*Hemophilus*	2	1.11
*Pseudomonas*	2	1.11
*Serratia*	2	1.11
*Acinetobacter*	1	0.55
*Adenovirus*	1	0.55
*BK virus*	1	0.55
*Candida*	1	0.55
*Clostridium*	1	0.55
*Cytomegalovirus*	1	0.55
*Escherichia* *coli*	1	0.55
*Group F Streptococcus*	1	0.55
*Human metapneumovirus*	1	0.55
*Moraxella*	1	0.55
*Parainfluenza*	1	0.55
*Rickettsia*	1	0.55
Unknown	74	40.88

### Identification of differentially expressed upregulated and downregulated genes

Based on preset criteria of an adjusted p-value < 0.05, a total of 53 genes from 21,731 were shown to be differentially expressed genes (DEG) between Survivor and Non-survivor types, including 47 genes that were upregulated and 7 genes that were downregulated. Those DEGs with a fold change of at least 1.5 (n = 16) is shown in Table 2 (For complete list, refer to Supplementary Dataset 1).

**Table 2:** List of most significant upregulated and downregulated genes in septic shock.

Gene	Fold Change	Average Expression	t-statistics	p-value	adj. p-value	Reference
*DDIT4*	2.12	8.441545	5.490723	1.33E-07	0.000592	^26,49^
*CCL3*	2.12	6.208106	4.627029	7.02E-06	0.012717	^29^
*PRG2*	2.11	5.48492	5.611777	7.35E-08	0.000533	—
*MT1M*	1.78	3.72997	5.641576	6.35E-08	0.000533	—
*CDC20*	1.68	6.18643	4.059008	7.31E-05	0.040893	^25^
*KIF20A*	1.66	4.940242	4.673723	5.74E-06	0.012717	—
*MAFF*	1.64	6.815799	4.437113	1.58E-05	0.015559	^50^
*EBI3*	1.64	5.41593	4.517058	1.12E-05	0.013575	^51^
*MELK*	1.63	6.413027	4.141523	5.27E-05	0.034718	—
*TOP2A*	1.58	4.997892	4.045113	7.72E-05	0.040893	^52^
*NUSAP1*	1.54	6.627514	3.928091	0.000121	0.049772	^53^
*RGL1*	1.52	7.288761	4.447937	1.51E-05	0.015559	^54^
*ARHGEF40*	−1.66	6.880613	−3.994983	9.38E-05	0.043368	—
*LOC254896*	−1.65	8.498379	−4.452352	1.48E-05	0.015558	—
*SLC46A2*	−1.61	5.880150	−4.084894	6.60E-05	0.039833	—
*TNFRSF10C*	−1.54	8.678817	−4.643272	6.55E-06	0.012717	^55^

### Functional enrichment analysis of differentially expressed genes

53 DEGs were analyzed by KEGG pathway and Gene Ontology (GO) term enrichment. A total of 52 genes were recognized in the DAVID database. KEGG pathway analysis revealed rheumatoid arthritis (RA) (hsa: 05323), and cell cycle (has: 04110) pathways as the most significant pathways (Table 3). GO analyses of the DEGs demonstrated that mitotic sister chromatid segregation (GO: 0000070), immune response (GO: 0006955), cell division (GO: 0051301), and chemokine-mediated signaling pathway (GO: 0070098) were the most enriched biological process (BP) terms (Table 3). Chemokine activity (GO: 0008009) was the most enriched term under molecular function (MF) (Table 3). Chemokine interleukin-8-like domain (IPR001811), CC chemokine, conserved site (IPR000827) InterPro protein functional groups were among the significantly enriched functional classes associated with septic shock development (Table 3).

**Table 3:** Functional enrichment of differentially expressed genes.

Functional Category	ID	Functional Term	Gene Count	adjusted p-value	Fold Change	Benjamini Score
BP	GO:0000070	Mitotic sister chromatid segregation	4	4.82E-05	54.83	0.0246
BP	GO:0006955	Immune response	8	1.80E-04	6.51	0.0453
BP	GO:0051301	Cell division	7	4.61E-04	6.85	0.0763
BP	GO:0070098	Chemokine-mediated signaling	4	0.001093	19.30	0.0776
BP	GO:0007059	Chromosome segregation	4	9.64E-04	20.15	0.0797
BP	GO:0007067	Mitotic nuclear division	6	6.88E-04	8.29	0.0851
BP	GO:0030593	Neutrophil chemotaxis	4	8.84E-04	20.76	0.0873
MF	GO:0008009	Chemokine activity	4	3.63E-04	28.12	0.0467
KEGG pathway	hsa05323	Rheumatoid arthritis	5	2.58E-04	15.03	0.0226
KEGG pathway	hsa04110	Cell cycle	5	9.49E-04	10.66	0.0413
InterPro	IPR001811	Chemokine interleukin-8-like domain	4	2.01E-04	34.33	0.0247
InterPro	IPR000827	CC chemokine, conserved site	3	0.001602	49.35	0.0953

BP: Biological Process; MF: Molecular Function; CC: Cellular Component.

**Figure 1:**
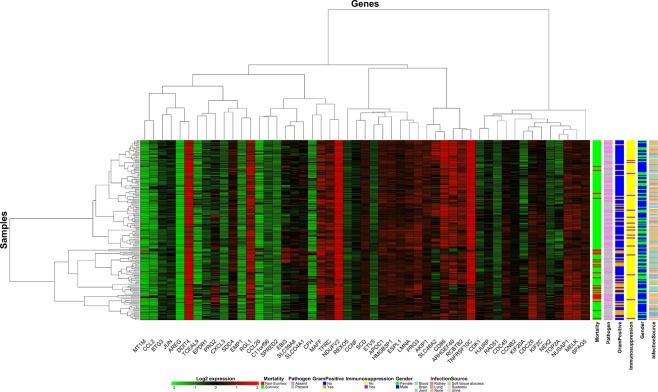
Heatmap representing differentially expressed genes between survivor and non-survivor groups, with pathogen, gram-positive, immunosuppression, gender status, and infection source as annotation.

Figure 1 illustrates a heatmap of 53 selected DEGs in survivor and non-survivor groups. The vertical axis represents patient samples (see Supplementary Dataset 1 for more details), and the horizontal axis represents DEGs with the log_2_ gene expression intensity values. Red and green represent the upregulated, and downregulated genes, respectively. The heatmap is clustered using both samples and genes categories. Right vertical bars are used for annotation of mortality, pathogen, gram-positive, immunosuppression, gender status, and infection source. There are two major sample clusters at the top level of the dendrogram. Only 4 (15%) out of 27 non-survivors were in the first cluster, while, the remaining 85% formed the second cluster. Similarly, for the gram-positive category, samples formed two clusters. We observed no clear trends or cluster formation for the other annotation groups. Genes were clustered into two major groups of downregulated genes (*MT1M* to *CFH* except for *DDIT4* and *RGL1*), while the remaining genes clustered in the upregulated category. Overall, *DDIT4*, *NDUFV2*, and *TNFRFS10C* were the most highly expressed genes, whereas *AREG*, *CCL20*, and *CFH* were the least expressed genes.

## Discussion

This study of peripheral blood mRNA sequences revealed key genes and functional characterization associated with septic shock survival and non-survival^23^. From the differential gene expression analysis, we identified potential septic shock biomarkers that may help in an unbiased septic shock diagnosis and effective treatment, and ultimately improving prognoses.

Differntially expressed gene analysis using septic shock samples provides insights into the functional characterization of genes between groups of septic shock survivor and non-survivor samples. However, like any other, microarray data analysis is incomplete without performing adjustment for multiple testing. Due to approximately 20,000 (approximate number of genes on a standard microarray chip) independent tests, it is expected to get at least 20 test scores by random chance when we allow a stricter p-value threshold of, for example 0.001. To avoid this situation, adjustment for multiple testing was utilized, and we used the *Benjamini Hochberg* method. For a false-discovery rate (FDR) controlling procedure, adjusted p-value of an individual hypothesis is the minimum value of FDR for which the hypothesis is first included in the set of rejected hypotheses, and we used an adjusted p-value cut-off of 0.05^24^.

We identified *CDC20* as one of the top upregulated genes, along with *LCN2*, and *CD24*, similar to the findings of Dong *et al*.^25^, which studied development of trauma-induced sepsis in patients. However, our study population was much more diverse (Table 2 and Supplementary Dataset 1). The most significant upregulated gene identified was *DDIT4* (DNA damage-inducible transcript 4-like). PERSEVERE-XP study had also identified *DDIT4* gene directly related to *TP53*^7^. *DDIT4* (*REDD1)* is increased in septic shock, can negatively regulate mTORC1 activity and plays an important role in energy homeostasis^26^. We found *CCL3* as the second-most significant upregulated chemokine, a fundamental component of acute-phase response to endotoxin in humans and regulation of leukocyte activation and trafficking^27^. Elevated levels of *CCL3* have been detected within the first 24 hours of sepsis, suggesting its unique role in innate immune function^28,29^. Further studies are needed to understand the mechanisms of these identified genes in septic shock development. On the other hand, *TNFRSF10C*, a downregulated gene has been shown to play an essential role in sepsis immune response^30^.

The set of genes identified is then examined for over-representation of specific functions or pathways. Septic shock survivors were distinguished from non-survivors by differential expression of genes involved in the immune response, chemokine-mediated signaling, neutrophil chemotaxis, and chemokine activity. Sepsis impacts the immune responses by directly altering life span, production, and function of effector cells responsible for homeostasis^31^. We identified the immune response (GO:0006955) term from DAVID analysis and there has also been evidence that understanding the immune response to sepsis provides opportunities to develop effective treatment strategies^32^.

Chemokines play a critical role in sepsis and septic shock development, and molecules that block chemokine and chemokine receptor activity may prove to be useful in the identification of sepsis^33^. Our differentially expressed genes mapped to chemokine-mediated signaling (GO:0070098), chemokine activity (GO:0008009) molecular function, chemokine interleukin-8 like domain (IPR001811), and chemokine conserved site (IPR000827).

Sepsis and rheumatoid arthritis (RA) have been known to be associated for over 50 years^34^. RA is shown to be a risk factor in sepsis patients and sepsis infection could trigger RA^35^. We identified RA KEGG pathway (hsa:05323) using our differentially expressed gene set to be statistically significant (Table 3). We analyzed the literature to look for other underlying genetic factors responsible for septic shock. Five (*CCL3, CDC20, TNFRSF10C, EBI3, TOP2A*) of the 53 identified genes were already shown to be involved in sepsis, and the remaining 48 genes from our list were not shown to be involved in sepsis. These genes may contribute to the septic shock response. Table 1 and the Discussion section above explains it in further detail. The *SLC46A2* is downregulated in the septic shock non-survivor group (Fig. 1) and have been shown to be involved in T-cell homeostasis (GO:0043029). On the other hand, the *LCN2* was identified as the upregulated gene and recently proposed as a biomarker involved in acute kidney injury (AKI)^36^. Overall, 22.6% (41/181) patients had AKI, whereas, in the non-survivors, there were 70% (19/27) patients who had AKI (see Supplementary Dataset 1 for more details).

Gene expression changes shown in our results are based on the peripheral blood cells and may not be extrapolated as occurring at the organ or tissue level^23,37^. Therefore, extra care must be taken while generalizing host immune responses or chemokine activities in septic shock patients. Besides, variations in gene expression profiles of survivors and non-survivors of septic shock patients could be due to other unexplored confounding factors (such as patient demographics) rather than sepsis-related biology^38^. On the other hand, the blood-based biomarkers have the advantage of being minimally-invasive. Therefore, in combination with other clinical phenotypic data, these minimally-invasive biomarkers may enable rapid recognition of poor long term outcomes^39^ Large cohorts replication studies and network analysis studies are needed to gain insights into relationships between these biomarkers and the survival/non-survival of cohorts^40^. To avoid the selection bias, analysis must be expanded to other independent data sets.

This work can be expanded by experimentally validating the identified blood-based biomarkers and developing robust machine learning methods to build a septic shock prediction models using different omics data from diversified patient cohorts.

## Materials and Methods

### Sepsis and septic shock

Bacteremia or another pathogenic infection triggers a serious inflammatory response called sepsis, which typically includes an increased number of white blood cells, rapid heart rate and breathing rate, and fever^1^. The response also affects many major organ failures. Sepsis that results in poor perfusion to end organs resulting in multiple organ failure is defined as a septic shock^7^.

### Data collection

Expression microarray data was collected from the NCBI Gene Expression Omnibus repository^41^. The dataset contains gene expression profiles of peripheral blood samples from 181 septic shock patients including 154 survivors and 27 non-survivors who were admitted to the pediatric intensive care unit (PICU) within the first 24 hours^42^. For all 181 samples, blood was drawn on day 1 of the PICU stay. GEO accession number for data used in the study is GSE66099. Data was collected from the Affymetrix Human Genome HG-U133_Plus_2 (GPL570 platform).

### Normalization and background correction

The R Affy module^43^ was used to remove technical variations and background noise. The Quantile Normalization Method^44^ was used to normalize data and background correction was performed using the Robust Multi-Average^45^ parameter method^46^.

### Probe to gene mapping

Affymetrix probes were mapped to the genes using information provided in the Affymetrix database (hgu133plus2.db). We used average expression values when multiple probes mapped to the same gene^19^.

### Identification of differentially expressed genes

Differentially Expressed Genes (upregulated and downregulated genes) were identified using R limma package with a Benjamini-Hochberg (BH) correction method and adjusted p-value of <0.05.

### Functional analysis

We used DAVID^47^ for functional enrichment analysis of the DEGs from samples of septic shock survivors or non-survivors. Biological Process (BP), Cellular Component (CC), and Molecular Function (MF) were identified from the Gene Ontology database. For the GO functional groups, KEGG pathways, and InterPro functional terms returned from DAVID functional analysis, we considered an adjusted p-value threshold of ≤0.05 and gene count of 3 or more from this study.

### Statistical analysis

R programming language^48^ was used for downloading the Affymetrix data and gene mapping using R Affy and Bioconductor package. A Fisher-exact test was performed for determining statistical significance among gene ontology terms and functional classes. Benjamini Hochberg multiple test correction method was used for calculating the differentially expressed genes. Heatmap of differentially expressed genes between survivor and non-survivor groups for top selected genes were plotted using the complexHeatmap function in R programming. Other variables (pathogen, gram-positive, immunosuppression, gender status, and infection source) were added to the heatmap for annotation.

## Supplementary information

Dataset 1

## Data Availability

R scripts other related files used for data preprocessing, normalization and differential gene expression analysis are available from https://github.com/akram-mohammed/septic_shock_degs. Datasets generated and analyzed during the study are available upon request.
